# Prediction of cardiovascular events by central blood pressure using radial tonometry in type 2 diabetes mellitus patients

**DOI:** 10.1186/s40885-022-00212-7

**Published:** 2022-10-15

**Authors:** Min-Sik Kim, Seon-Ah Cha, Gee-Hee Kim

**Affiliations:** 1 Division of Cardiology, Department of Internal Medicine, College of Medicine, St. Vincent’s Hospital, The Catholic University of Korea, Seoul, Republic of Korea; 2 Division of Endocrinology and Metabolism, Department of Internal Medicine, College of Medicine, St. Vincent’s Hospital, The Catholic University of Korea, Seoul, Republic of Korea; 3 College of Medicine, Catholic Research Institute for Intractable Cardiovascular Disease, The Catholic University of Korea, Seoul, Republic of Korea

**Keywords:** Hypertension, Central blood pressure, Atherosclerotic cardiovascular disease risk assessment

## Abstract

**Background:**

High blood pressure (BP) and type 2 diabetes mellitus (T2DM) are major causes of atherosclerotic cardiovascular disease (ASCVD) and heart failure (HF). Central blood pressure (CBP) is more predictive of ASCVD than is brachial BP; however, an association of CBP with ASCVD has not been found in T2DM patients. We evaluated the impact of CBP and the association between optimal level of noninvasively measured CBP and office BP in T2DM patients based on composite outcome of ASCVD, HF, and complications of hypertension.

**Methods:**

Patients were enrolled from June 2011 to December 2015 and were followed up through December 2019. CBP was measured using radial tonometry. The primary endpoints were composite outcome of ASCVD, HF, and hypertension-induced complications such as left ventricular hypertrophy, retinopathy, and proteinuria.

**Results:**

During the 6.5-year follow-up period, 515 patients were enrolled in the study. A total of 92 patients (17.9%) developed primary endpoints. The mean age of subjects was 61.3 ± 12.1 years and 55% (*n* = 283) were male. Patients who developed primary endpoints were older (65.3 ± 9.5 years vs. 60.5 ± 12.4 years) and had lower high-density lipoprotein (36.6 ± 9.4 mg/dL vs. 41.8 ± 11.1 mg/dL), higher CBP (123.6 ± 20.6 mmHg vs. 118.0 ± 20.6 mmHg), and higher pulse pressure (61.3 ± 16.6 mmHg vs. 56.5 ± 15.1 mmHg) than subjects without primary endpoint development. After adjustment for various risk factors, CBP was an independent predictor for primary endpoints (hazard ratio, 1.14; 95% confidence interval, 1.02–1.27; *P* = 0.016). In addition, the association of CBP and primary endpoints showed a U-shaped curve with the lowest incidence at CBP 118 mmHg and systolic BP about 128 mmHg.

**Conclusions:**

We show the importance of CBP measurements in T2DM patients and present a cutoff value for ASCVD events and hypertension-induced complications.

## Background

The number of people with hypertension has increased worldwide from 650 million to 1.28 billion in the last 30 years [1]. High blood pressure (HBP) can cause asymptomatic damage to the heart, such as left ventricular hypertrophy (LVH). Subsequently, the risk of atherosclerotic cardiovascular disease (ASCVD) is reported to increase the risk of angina by 1- to sixfold, myocardial infarction (MI) by 2- to fivefold, and stroke by 3- to tenfold [2–5]. It is also reported that cardiovascular (CV) mortality gradually increases with every 20 mmHg increase in systolic BP (SBP), while lower mortality from ischemic heart disease (7%) and lower stroke mortality (about 10%) are found with every 2 mmHg decrease in average SBP [6].

HBP is a strong risk factor for ASCVD in patients with type 2 diabetes mellitus (T2DM) [7]. In a recent large, primary care-based cohort study of Swedish patients with T2DM, the association of SBP with risk of CV events and mortality showed a U-shaped curve in patients both with and without regular use of antihypertensive drugs [8]. In clinical practice guidelines, it is recommended that BP targets should be individualized according to CV risk and should be lower in patients with ASCVD than in patients without ASCVD in T2DM [9]. However, the appropriate BP cutoff in patients with T2DM for preventing CV events is unclear.

Several studies have shown that central BP (CBP) [10, 11], which is the pressure measured from the central aorta or common carotid arteries, might be superior to brachial BP (BBP) in the prediction of ASCVD events and target organ damage, although BBP conventionally measured in daily office practice and used to diagnose HBP [12]. However, SBP may be up to 40 mmHg higher in the brachial artery than in the aorta, although diastolic and mean arterial pressures are relatively constant [13]. This phenomenon of systolic pressure amplification arises principally because of an increase in arterial stiffness as the distance from the heart increases. Also, the discrepancy between CBP and BBP is purported to be influenced by numerous demographic and physiological factors including age, sex, and heart rate [14, 15]. However, in patients with T2DM, the impact of CBP level measured by radial pulse wave velocity (PWV) on CV risk assessment and the relationship between CBP and BBP are unclear. Thus, this study intends to present the effectiveness and cutoff value of CBP as a predictive factor of major adverse CV events and hypertension-induced complications in T2DM patients.

## Methods

### Study population

An initial cohort of participants with T2DM who presented with or without concomitant CV risk factors or target organ damage was selected from the Department of Internal Medicine, St. Vincent’s Hospital, from July 2011 to December 2015. Among the patients who underwent noninvasive, semiautomated, radial artery applanation tonometry (Omron HEM-9010AI, Omron Healthcare, Kyoto, Japan) and were eligible for our study, 515 (283 male patients, 55%; mean age, 61.3 ± 12.1 years) were enrolled in this study. Based on self-reported menopause state, female patients were classified as premenopausal or postmenopausal. Exclusion criteria were clinical or laboratory findings of acute CV events within 3 months prior to enrollment. Subjects who had an irregular cardiac rhythm or brachial artery stenosis were excluded due to the method used to measure radial PWV.

There was no industry involvement in the design, implementation, or data analysis of this study. The present study was a single-center retrospective study and was approved by the Institutional Review Board of St. Vincent’s Hospital (VC21RISI0202).

### Measurement of brachial blood pressure and central blood pressure

Participants rested for at least 5 min in a quiet room and were seated comfortably with their legs uncrossed and their back and arms supported. BBP was measured using an automatic cuff oscillometric device (HEM907, Omron Healthcare). The average of three readings was used to determine SBP mean arterial pressure, and pulse pressure (PP) [11]. Next, the radial pulse wave was obtained with an automated applanation tonometer (HEM-9010AI). The method to measure CBP was the same as in a previous study [16].

### Clinical and biochemical assessments

Blood specimens were obtained after a 12- to 14-h fast (8:00 PM to 9:30 AM) to reduce the influence of circadian variation. Total cholesterol [17] and triglyceride [8] concentrations were assessed using standard enzymatic methods. High-density lipoprotein (HDL) cholesterol level was measured after precipitation of very-low-density lipoproteins and low-density lipoproteins (LDL) with phosphotungstic acid, and LDL was calculated using the Friedewald formula. Serum samples were stored at –80 °C, and high-sensitivity C-reactive protein was determined using an immunoturbidity assay (Liatest; Stago, Asnieres-sur-Seine, France), with a 6.25% interassay variability coefficient.

### Outcomes

The primary endpoints were composite outcome of ASCVD events or death from ASCVD and hypertension-induced complications, including newly diagnosed atrial fibrillation (AF), heart failure (HF), and HBP complication such as LVH, retinopathy, and proteinuria. ASCVD was defined as the presence of acute coronary syndrome (ACS, including ST elevation MI, non-ST elevation MI, and unstable angina) or a history of MI, stable or unstable angina, coronary or other arterial revascularization, cardiorovascular disease (CVD) including stroke or transient ischemic attack, or peripheral arterial disease (PAD) defined as an ankle-brachial index < 0.9 measured using an Omron VP-1000 Vascular Profiler (Omron Healthcare) presumed to be of atherosclerotic origin. Medical records were obtained from ASCVD-related physician visits during follow-up and were reviewed by cardiologists.

### Statistical analyses

Continuous variables are presented as mean ± standard deviation, and categorical variables are presented as absolute and relative frequencies (%). A t-test was used to compare the means between two groups. Proportions were compared using two-way tables and chi-square tests. To identify the independent predictors of primary endpoints, multivariate analyses using the Cox proportional hazard regression model were applied to the variables that were significant in univariate analysis and were known important risk factors for primary endpoints. Two-sided ≤ 0.05 indicate statistical significance. Multivariate analyses were schematized using a restricted cubic spline curve. All statistical analyses were conducted using R ver. 3.6.3 (The R Foundation for Statistical Computing, Vienna, Austria; https://www.r-project.org/).

## Results

The median follow-up period was about 6.5 years, with an average patient age of 61.3 ± 12.1 years and 55% of patients were male. A total of 92 patients (17.9%) developed primary endpoints during the follow-up period. Of the 92 events, 52 were ACS (57%), 14 were CVA (15%), 3 were PAD (3%), 8 were HF events (9%), hypertension complications were found in 12 cases (13%), and AF were cases (3%). Table 1 shows patient groups by cumulative incidence of primary endpoints. Mean body mass index (BMI) was 24.8 ± 3.4 kg/m^2^, and the median T2DM duration was about 9.0 years, with 99.8% of patients using oral hyperglycemic agents and 24.6% using insulin. Regarding patient history, 64.9% of patients had HBP and 67.4% of patients had previously taken antihypertensive drugs. The average HbA1c was 7.8% ± 1.9%. As seen in Table 1, age, T2DM duration, CBP, SBP, and renal function showed significant differences in the occurrence of primary endpoints.

**Table 1:** Baseline characteristics of the participants

Baseline characteristics	Overall (*n* = 515)	Primary endpoint (–) (*n* = 423)	Primary endpoint ( +) (*n* = 92)	*P*-value
Age (yr)	61.34 ± 12.1	60.48 ± 12.4	65.34 ± 9.5	< 0.001
Male sex	283 (55.0)	231 (54.6)	52 (56.5)	0.827
Body mass index (kg/m^2^)	24.78 ± 3.4	24.72 ± 3.5	25.05 ± 3.2	0.395
Smoking	171 (33.6)	140 (33.4)	31 (34.4)	0.948
Diabetes complication	175 (34.0)	144 (34.0)	31 (33.7)	1.000
Diabetes duration (yr)	8.95 ± 8.7	8.56 ± 8.5	10.69 ± 9.6	0.044
HBP	334 (64.9)	271 (64.1)	63 (68.5)	0.495
CAD	209 (85.7)	149 (83.7)	60 (90.9)	0.222
Laboratory measurements				
Fasting blood glucose (mg/ dL)	159.38 ± 68.1	158.36 ± 67.1	164.00 ± 72.8	0.490
HbA1c (%)	7.82 ± 1.9	7.84 ± 1.9	7.74 ± 1.5	0.682
C-reactive protein (mg/dL)	0.82 ± 2.6	0.79 ± 2.6	0.97 ± 2.6	0.654
eGFR (mL/min/1.73m^2^)	90.71 ± 29.8	92.38 ± 30.4	83.11 ± 25.8	0.008
Total cholesterol (mg/dL)	173.68 ± 43.4	174.12 ± 42.6	171.67 ± 47.4	0.639
Triglyceride (mg/dL)	150.93 ± 126.6	146.49 ± 113.0	171.04 ± 174.8	0.108
LDL (mg/dL)	100.97 ± 34.8	101.58 ± 35.2	98.24 ± 32.9	0.427
HDL (mg/dL)	40.83 ± 10.9	41.76 ± 11.0	36.62 ± 9.4	< 0.001
Medication				
Oral hyperglycemic agent	446 (99.8)	370 (100.0)	76 (98.7)	0.385
Insulin	110 (24.6)	93 (25.1)	17 (22.1)	0.674
Lipid lowering agent	248 (48.2)	199 (47.0)	49 (53.3)	0.334
Premedication	347 (67.4)	276 (65.2)	71 (77.2)	0.037
ACEi, ARB	183 (39.4)	148 (38.8)	35 (41.7)	0.722
Beta blocker	109 (23.4)	83 (21.8)	26 (31.0)	0.098
Calcium channel blocker	129 (27.7)	103 (27.0)	26 (31.0)	0.554
Diuretics	85 (18.3)	63 (16.5)	22 (26.2)	0.055
Baseline characteristics	Overall	Primary endpoint (–)	Primary endpoint ( +)	P-value
CBP (mmHg)	118.98 ± 20.7	117.98 ± 20.6	123.60 ± 20.6	0.018
SBP (mmHg)	131.05 ± 19.9	130.22 ± 19.7	134.86 ± 20.3	0.042
DBP (mmHg)	73.71 ± 12.2	73.75 ± 12.6	73.53 ± 9.9	0.874
Pulse pressure (mmHg)	57.34 ± 15.5	56.47 ± 15.1	61.33 ± 16.6	0.006
Augmentation index	79.75 ± 13.3	79.36 ± 13.2	81.50 ± 13.8	0.163
Heart rate	75.42 ± 13.3	75.50 ± 13.2	75.04 ± 13.9	0.775

Data are presented as mean ± standard deviation or number (%)

*HBP* High Blood Pressure, *CAD* Coronary Artery Disease, *eGFR* estimated Glomerular Filtration Rate, *LDL* Low-density Lipoprotein, *HDL* Highdensity Lipoprotein, *ACEi* Angiotensin Converting Enzyme inhibitor, *ARB* Angiotensin Receptor Blocker, *CBP* Central Blood Pressure, *SBP* Systolic Blood Pressure, *DBP* Diastolic Blood Pressure

In Table 2, univariate Cox regression for each variable is shown. In univariate analysis, age (hazard ratio [HR], 1.04; 95% confidence interval [CI], 1.02–1.06; P < 0.001), renal function (HR, 0.99; 95% CI, 0.98–1.00; *P* = 0.002), HDL (HR, 0.95; 95% CI, 0.93–0.98; P < 0.001), and longer diabetes duration (HR, 1.03; 95% CI, 1.00–1.05; *P* = 0.031) showed a significant increase of primary endpoints. In the incidence of primary endpoints, there was no significant difference according to sex; however, postmenopausal women were significantly more frequent than men (HR, 1.02; 95% CI, 1.01–1.03, *P* = 0.005). In multivariate analysis, CBP was a significant predictor for primary endpoints (HR 1.01; 95% CI 1.01–1.02, *P* = 0.029) after adjustment for age, sex, smoking, BMI, and T2DM complications (Table 3). And when CBP was grouped and analyzed to increase every 10 mmHg, the role as a significant predictor has become clearer. In univariate Cox regression, HR was 1.17 (95% CI, 1.06–1.30; *P* = 0.002) and HR was 1.14 (95% CI, 1.02–1.27; *P* = 0.016) even when age, gender, smoking, and diabetes complications were adjusted.

**Table 2:** Univariate Cox regression

Variable	Hazard ratio	95% Confidence interval	*P*-value
Age	1.04	1.02–1.06	< 0.001
Sex			
Male	1.04	0.69–1.58	0.835
Female (premenopausal)	1.00	0.97–1.03	0.991
Female (postmenopausal)	1.02	1.01–1.03	0.005
Body mass index	1.02	0.97–1.08	0.405
Smoking	1.08	0.69–1.67	0.739
Diabetes complication	0.91	0.59–1.41	0.687
Diabetes duration	1.03	1.00–1.05	0.031
High blood pressure	1.33	0.85–2.07	0.215
Coronary artery disease	2.12	0.91–4.96	0.082
HbA1c	0.95	0.83–1.09	0.454
C-reactive protein	1.00	0.94–1.10	0.576
Estimated glomerular filtration rate	0.99	0.98–1.00	0.002
Total cholesterol	1.00	0.99–1.00	0.610
Lowdensity lipoprotein	1.00	0.99–1.01	0.705
High-density lipoprotein	0.95	0.93–0.98	< 0.001
Insulin	0.78	0.45–1.36	0.454
Lipid lowering agent	1.01	0.74–1.70	0.608
Premedication	1.67	1.02–2.74	0.043

**Table 3:** Univariate and multivariate Cox regression

Variable	Univariate analysis			Multivariate analysis		
	HR	95% CI	*P*-value	HR	95% CI	*P*-value
CBP	1.01	1.01–1.02	0.003	1.01	1.01–1.02	0.029
CBP (per 10 mmHg)	1.17	1.06–1.30	0.002	1.14	1.02–1.27	0.016
SBP	1.01	1.01–1.02	0.003	1.01	1.01–1.02	0.050
DBP	1.00	0.98–1.02	0.918	1.01	0.99–1.03	0.409
Pulse pressure	1.02	1.01–1.03	< 0.001	1.01	1.01–1.03	0.049
Augmentation index	1.01	1.01–1.03	0.090	1.01	0.99–1.03	0.325

Adjustment factors are age, body mass index, smoking, and diabetes mellitus complication

*HR* Hazard Ratio, *CI* Confidence Interval, *CBP* Central Blood Pressure, *SBP* Systolic Blood Pressure, *DBP* Diastolic Blood Pressure

**Fig. 1:**
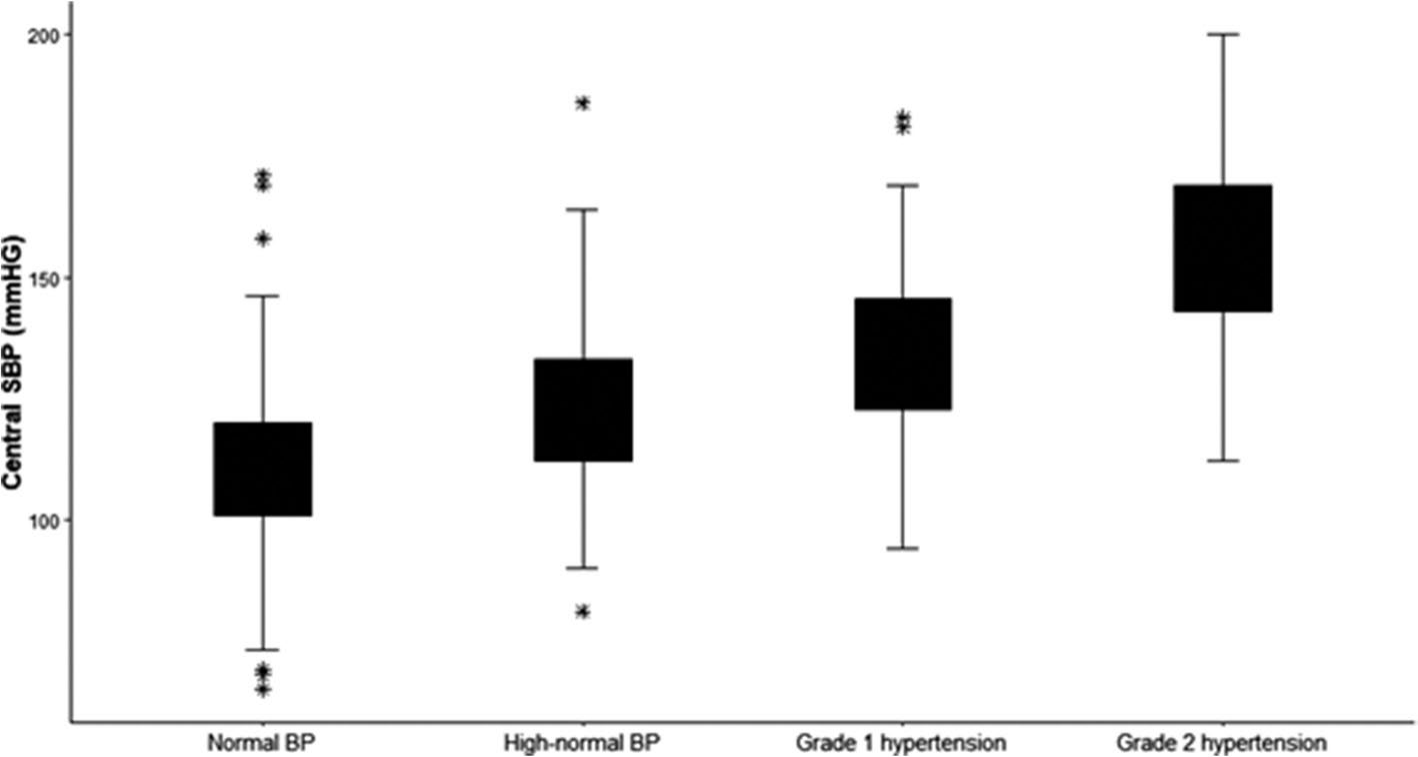
Linear correlation between central blood pressure (BP) and systolic BP (SBP). Normal BP, < 130 mmHg; high-normal BP, 130–139 mmHg; grade 1 hypertension, 140–159 mmHg; grade 2 hypertension, ≥ 160 mmHg. Statistical analyses were conducted using R ver. 3.6.3 (The R Foundation for Statistical Computing, Vienna, Austria; https://www.r-project.org/)

**Fig. 2:**
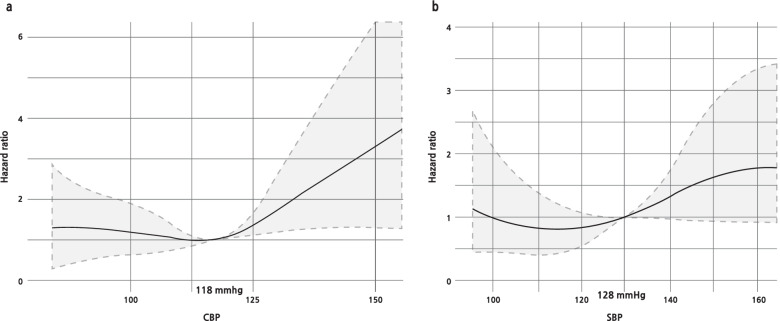
Restricted cubic spline curve of **A** central blood pressure (CBP) and **B** systolic BP (SBP). Based on each multivariate cox regression model, the lowest hazard ratio in CBP was 118 mmHg and 128 mmHg in SBP. Statistical analyses were conducted using R ver. 3.6.3 (The R Foundation for Statistical Computing, Vienna, Austria; https://www.r-project.org/)

As shown in Fig. 1, a linear correlation between CBP and BBP was confirmed. The analysis of CBP as a restricted cubic spline curve with outcome to ASCVD events is shown in Fig. 2A. The incidence of primary endpoints tended to increase as CBP increased, and the incidence of primary endpoints increased even at lower CBP. The lowest incidence of primary endpoints was seen at CBP 118 mmHg. Figure 2B shows the analysis of SBP as a restricted cubic spline curve with primary endpoints. Similar to previous CBP results, the incidence of primary endpoints tended to increase as SBP increased or decreased, and the lowest incidence of primary endpoints at SBP was at about 128 mmHg.

## Discussion

This study found that CBP is a clinically significant predictor for primary endpoints, showing a U-shaped association of CBP and risk of CV events in T2DM. The lowest incidence of primary endpoints was seen at CBP of 118 mmHg and SBP of about 128 mmHg.

A previous large cohort study reported an association of SBP and risk of CV events and mortality, with a U-shaped curve pattern in T2DM [8], and SBP was found to have the lowest incidence at 135–139 mmHg the manual Korotkoff method or automatic measurement. In a study conducted in the general population [18], CBP had a threshold of 112 mmHg and SBP of 121 mmHg when a digital automatic BP monitor was used; however, the study did not show a U-shaped pattern. The CBP of 118 mmHg and SBP of 128 mmHg found in our study of the T2DM population are more stringent than those used in previous studies of T2DM patients (SBP, 135–139 mmHg) and are less intensive than those in the general population (CBP, 112 mmHg and SBP, 121 mmHg), which might require more tight objectives than previously considered by T2DM patients. However, in comparison with the general population, optimal CBP in T2DM patients can suggest increased risk of CV events. These assumptions are confirmed as a result.

In the result, risk ratio to an increase in CBP of 10 mmHg. As a result, this study suggests that after 6.5 years of follow-up, the risk of CVA increases by 14% for every 10 mmHg increase in CBP (HR, 1.14 *P* = 0.016).

In addition, previous studies suggested that there is a limitation in providing accurate information about the patient's BP status due to the problem of variability in peripheral BP. Although somewhat cumbersome, noninvasively measured central pulse pressure is more strongly associated with vasoconstriction, severity of atherosclerosis, and CV events than BBP as is known from the Strong Heart Study. Therefore, it is thought that measuring CBP may be important in assessing each patient's CV risk in order to more accurately determine the patient's BP status. So optimal CBP setting is more important in T2DM patients than general population.

A previous study conducted in the general population confirmed an increase in SBP in a cascading manner as CBP increased [19] and showed that CBP levels overlap significantly in hypertensive patients classified as SBP. In this study, SBP was increased stepwise in CBP in the T2DM population, suggesting that patients currently classified as hypertensive and receiving the same treatment might need better control through CBP measurement. A mechanism that explains the CBP and CV events in T2DM has been proposed. CBP is a stronger stimulus for LVH than is peripheral BP. Aortic stiffness also might be associated with carotid flow index, which contributes to altered flow dynamics resulting in increased CV risk [20]. In addition, T2DM patients are exposed to CV risk factors including hyperglycemia, advanced glycation end products, and diabetes duration [21], which lead to increased risk of CVA in T2DM. A meta-analysis also showed that markers of central systolic load were significantly increased in T2DM compared to those without T2DM, which could not be identified by BBP [5]. This difference might be associated with demographic or physiologic factors including age, sex, BMI, heart rate, and antihypertensive medication [15, 22].

Postmenopausal female patients showed a greater risk of study end point than male patients. These findings were similar to a study conducted in the Korean population, confirming that central PP tended to be higher in men before 3847 years of age, but the slope was steeper in women than in men at later ages [23]. This is thought to be due to the vascular protective effect of estrogen, as confirmed in previous studies [24], but cannot be explained simply by hormones as some studies suggest that risk is greater than benefit [25].

This study has several limitations. First, the study design is observational and retrospective; consequently, we could not control all confounding factors that affect ASCVD events or hypertension-induced complications. We attempted to adjust confounding factors to reduce this effect. Second, this study included only Korean subjects. In addition, the normal range or cutoff value of CBP has not been confirmed. This study also has plausible strengths in that it is the largest study of CBP measurement, CV events, and hypertension-induced complications and was conducted with long-term follow-up in patients with T2DM.

## Conclusions

With the logic that CBP may be more efficient than peripheral BP in predicting ASCVD, this study evaluated the risk of ASCVD in diabetic patients through CBP measurements. Accordingly, as confirmed in previous studies, the U-shape pattern, which increases the risk when BP increases and decreases, was confirmed, and the lowest HR was suggested as cutoff of 118 mmHg in CBP and 128 mmHg in SBP. In addition, it is thought that this will require research on the clinical importance and cutoff of CBP and SBP in various populations, and clinical application thereof may be necessary.

## Data Availability

The datasets used and/or analyzed during the current study are available from the corresponding author on reasonable request.

## Abbreviations

ACEi: Angiotensin converting enzyme inhibitor
ACS: Acute coronary syndrome
ARB: Angiotensin receptor blocker
ASCVD: Atherosclerotic cardiovascular disease
AF: Atrial fibrillation
BBP: Brachial blood pressure
BMI: Body mass index
BP: Blood pressure
CV: Cardiovascular
CBP: Central blood pressure
CVD: Cardiovascular disease
CI: Confidence interval
DBP: Diastolic blood pressure
eGFR: Estimated glomerular filtration rate
HBP: High blood pressure
HDL: High-density lipoprotein
HF: Heart failure
HR: Hazard ratio
LDL: Low-density lipoproteins
LVH: Left ventricular hypertrophy
MI: Myocardial infarction
PAD: Peripheral arterial disease
PP: Pulse pressure
PWV: Pulse wave velocity
SBP: Systolic blood pressure
T2DM: Type 2 diabetes mellitus

## References

[1] NCD Risk Factor Collaboration (NCD-RisC). Worldwide trends in hypertension prevalence and progress in treatment and control from (1990). to 2019: a pooled analysis of 1201 population-representative studies with 104 million participants. Lancet.

[2] Burke AP, Farb A, Liang YH, Smialek J, Virmani R (1996). Effect of hypertension and cardiac hypertrophy on coronary artery morphology in sudden cardiac death. Circulation.

[3] Bombelli M, Facchetti R, Carugo S, Madotto F, Arenare F, Quarti-Trevano F (2009). Left ventricular hypertrophy increases cardiovascular risk independently of in-office and out-of-office blood pressure values. J Hypertens.

[4] Levy D, Garrison RJ, Savage DD, Kannel WB, Castelli WP (1990). Prognostic implications of echocardiographically determined left ventricular mass in the Framingham Heart Study. N Engl J Med.

[5] Climie RE, Schultz MG, Fell JW, Romero L, Otahal P, Sharman JE (2019). Central-to-brachial blood pressure amplification in type 2 diabetes: a systematic review and meta-analysis. J Hum Hypertens.

[6] Lewington S, Clarke R, Qizilbash N, Peto R, Collins R, Prospective Studies Collaboration (2002). Age-specific relevance of usual blood pressure to vascular mortality: a meta-analysis of individual data for one million adults in 61 prospective studies. Lancet.

[7] Patel A, MacMahon S, Chalmers J, Neal B, Woodward M, ADVANCE Collaborative Group (2007). Effects of a fixed combination of perindopril and indapamide on macrovascular and microvascular outcomes in patients with type 2 diabetes mellitus (the ADVANCE trial): a randomised controlled trial. Lancet.

[8] Sundström J, Sheikhi R, Ostgren CJ, Svennblad B, Bodegård J, Nilsson PM (2013). Blood pressure levels and risk of cardiovascular events and mortality in type-2 diabetes: cohort study of 34 009 primary care patients. J Hypertens.

[9] American Diabetes Association (2021). 10. Cardiovascular disease and risk management: standards of medical care in diabetes-2021. Diabetes Care.

[10] Pickering TG, Hall JE, Appel LJ, Falkner BE, Graves J, Hill MN (2005). Recommendations for blood pressure measurement in humans and experimental animals: part 1: blood pressure measurement in humans: a statement for professionals from the subcommittee of professional and public education of the American heart association council on high blood pressure research. Hypertension.

[11] Cheng HM, Chuang SY, Wang TD, Kario K, Buranakitjaroen P, Chia YC (2020). Central blood pressure for the management of hypertension: is it a practical clinical tool in current practice?. J Clin Hypertens (Greenwich).

[12] Lee HY, Shin J, Kim GH, Park S, Ihm SH, Kim HC (2019). 2018 Korean society of hypertension guidelines for the management of hypertension: part II-diagnosis and treatment of hypertension. Clin Hypertens.

[13] Ohte N, Saeki T, Miyabe H, Sakata S, Mukai S, Hayano J (2007). Relationship between blood pressure obtained from the upper arm with a cuff-type sphygmomanometer and central blood pressure measured with a catheter-tipped micromanometer. Heart Vessels.

[14] McEniery CM, Yasmin N, Hall IR, Qasem A, Wilkinson IB, Cockcroft JR (2005). Normal vascular aging: differential effects on wave reflection and aortic pulse wave velocity: the Anglo-Cardiff Collaborative Trial (ACCT). J Am Coll Cardiol.

[15] Albaladejo P, Copie X, Boutouyrie P, Laloux B, Déclère AD, Smulyan H (2001). Heart rate, arterial stiffness, and wave reflections in paced patients. Hypertension.

[16] Kim G, Kim JH, Moon KW, Yoo KD, Ihm SH, Youn HJ (2014). The clinical usefulness of central hemodynamics to evaluate diastolic dysfunction in subjects without hypertension. Clin Interv Aging.

[17] Fryar CD, Ostchega Y, Hales CM, Zhang G, Kruszon-Moran D (2017). Hypertension prevalence and control among adults: United States, 2015–2016. NCHS Data Brief.

[18] Lamarche F, Agharazii M, Madore F, Goupil R (2021). Prediction of cardiovascular events by type I central systolic blood pressure: a prospective study. Hypertension.

[19] Sharman JE, Fang ZY, Haluska B, Stowasser M, Prins JB, Marwick TH (2005). Left ventricular mass in patients with type 2 diabetes is independently associated with central but not peripheral pulse pressure. Diabetes Care.

[20] Hashimoto J, Ito S (2013). Aortic stiffness determines diastolic blood flow reversal in the descending thoracic aorta: potential implication for retrograde embolic stroke in hypertension. Hypertension.

[21] Hegab Z, Gibbons S, Neyses L, Mamas MA (2012). Role of advanced glycation end products in cardiovascular disease. World J Cardiol.

[22] Vergnaud AC, Protogerou AD, Li Y, Czernichow S, Vesin C, Blacher J (2008). Pulse pressure amplification, adiposity and metabolic syndrome in subjects under chronic antihypertensive therapy: the role of heart rate. Atherosclerosis.

[23] Lee HY, Oh BH (2010). Aging and arterial stiffness. Circ J.

[24] Iorga A, Cunningham CM, Moazeni S, Ruffenach G, Umar S, Eghbali M (2017). The protective role of estrogen and estrogen receptors in cardiovascular disease and the controversial use of estrogen therapy. Biol Sex Differ.

[25] Rossouw JE, Anderson GL, Prentice RL, LaCroix AZ, Kooperberg C, Stefanick ML (2002). Risks and benefits of estrogen plus progestin in healthy postmenopausal women: principal results from the women’s health initiative randomized controlled trial. JAMA.

